# Using Laser Therapy and Topical Ozone as an Effective Intervention to Revolutionize Denture Stomatitis Treatment: A Case Report

**DOI:** 10.7759/cureus.62795

**Published:** 2024-06-20

**Authors:** Gunmeek Kaur, Prasanna R Sonar, Ankita Pathak, Sanja S Sahu

**Affiliations:** 1 Oral and Maxillofacial Surgery, Luxmi Bai Dental College and Hospital, Patiala, IND; 2 Oral Medicine and Radiology, Sharad Pawar Dental College and Hospital, Datta Meghe Institute of Higher Education and Research (Deemed to be University), Wardha, IND; 3 Prosthodontics, Sharad Pawar Dental College and Hospital, Wardha, IND; 4 Conservative Dentistry and Endodontics, Kalinga Institute of Dental Sciences, Kalinga Institute of Industrial Technology (Deemed to be University), Bhubaneshwar, IND

**Keywords:** ozonated olive oil, diode laser, denture stomatitis, candida albicans, oral hygiene

## Abstract

In dentistry, denture stomatitis (DS), a frequent inflammatory illness of the oral mucosa mostly related to denture wearing, is a major concern. DS is a common oral mycotic infection for those who wear partial or total dentures. The most often found species of *Candida* that may be isolated from both healthy and diseased oral tissues is *Candida albicans*. DS is associated with uneven denture surfaces, inadequate oral hygiene, or ill-fitting dentures. The diagnosis and management of DS in a 72-year-old male patient is presented in this case study. The patient complained about burning in his palate and having difficulty chewing. Upon clinical examination, erythema and inflammation were seen in the palate region. The history and clinical findings were consistent with DS. The patient was told to completely stop using dentures. Interventions were included in the treatment plan, such as diode laser therapy, topical ozonated oil application, and teaching about denture hygiene. The third day's follow-up visit revealed a progressive healing of the lesions and symptom relief. The lesion fully resolved on the sixth day. This case emphasizes the value of all-encompassing management techniques in treating DS successfully. It also highlights the significance of patient education, good oral hygiene, and focused therapy in producing favorable results.

## Introduction

The oral mucosal tissues covered by dentures can become inflamed and erythematous due to a common condition known as denture stomatitis (DS) [1]. DS is often referred to as prosthetic stomatitis and denture sore mouth. DS can result from morphologic changes such as hyperplasia, traumatic ulceration, or histopathologic changes including inflammation and degeneration of the soft tissues beneath the dentures. Its prevalence rises with age, and its frequency of development ranges from 25% to 67%, primarily in women. According to epidemiological research, denture wearers may have a prevalence of DS ranging from 15% to over 70% [1-5]. Partial or complete denture patients exhibit heightened vulnerability to DS due to factors such as prolonged denture wear, frequently neglected oral hygiene, a propensity for nutritional and immunological deficiencies, systemic diseases, use of antibiotics or corticosteroids, xerostomia prevalence, and general weakness [6].

The accumulation of dental plaque, continuous wearing of detachable dentures at night, inadequate denture cleanliness, and bacterial and yeast contamination of the denture surface are etiological factors and associated risk factors. Additionally, mucosal damage may occur if dentures are not fitted properly. All of these elements make it easier for *Candida albicans* to colonize oral mucosal surfaces and denture surfaces, where it functions as an opportunistic pathogen [1]. A significant etiologic factor in chronic candidiasis is yeast adhering to the prosthesis. There may be a reason for the severe candidal infection in patients with xerostomia since mucus and serum increase the adhesion of yeast to the patient's prosthesis, whereas salivary pellicle decreases it. Denture soft liners offer a porous surface and a way for plaque and yeast to be mechanically bound to the appliance. The common cause of DS and colonization is inadequate denture hygiene practices. *Candida albicans* colonization has been found in conjunction with bacteria or solely on the fitting surface of prostheses [7]. Although *Candida albicans *is a commensal microbe in the oral cavity of 45%-65% of normally healthy people, it is frequently linked with DS; however, denture wearers have a prevalence of up to 60%-100% [8]. Other associated factors can be local or systemic. Systemic factors include endocrine dysfunction, malnutrition, neoplasias, immunosuppression, autoimmune disease, broad-spectrum antibiotics, and topical or inhaled corticosteroids. Local variables include poor denture fitting, poor oral hygiene, a diet high in carbohydrates, increased alcohol and tobacco use, decreased salivary flow, an ill-fitting denture, and wearing the denture constantly-especially at night. This illness may also be exacerbated by an excessive amount of trauma [4,9-11]. The severity of DS symptoms varies, ranging from discomfort and irritation to minimal symptoms. Sometimes, a significant overgrowth of *Candida *can cause discomfort, changes in taste, dysphagia, and a burning feeling in the oral cavity [4]. Newton [4,10] classified the condition into three clinical categories: type 1 (point-blank hyperemia) and type 2 (generalized erythema), which is the most prevalent form of chronic candidiasis manifests as diffuse erythema and edema of the palatal mucosa areas that support dentures. At the denture's edges, the afflicted mucosa is either bright red or dark and can be easily distinguished from the surrounding mucosa. Most patients do not report pain, although angular cheilitis is typically present in conjunction with the illness. The granular type (inflammatory papillary hyperplasia) is represented by type 3 DS.

The majority of DS cases require a complete treatment strategy, which is started by determining the risk factors [12,13]. Consequently, clinicians should always begin with denture defect eradication, denture plaque control recommendations, and discontinuous denture wear at night [7]. Ozone's ability to oxidize serves as the foundation for its biocidal properties against bacteria, viruses, and fungi. According to reports, ozonizing oil does not change the ozone's medicinal qualities or maintain its stable state. Numerous superficial infections, including those brought on by *Candida*, have been reported to respond well to ozone treatment [12-14]. An infection will typically go away if proper oral and denture care is maintained and an ill-fitting denture is corrected [13-15]. Treatments for infections, particularly those of types 1 and 2, include laser beam, cryosurgery, electrosurgery, and scalpel surgery [4,16,17]. It is advised against using antifungal therapy or to save it for patients who are severely ill or who have impaired systems due to side effects and potential intolerance [4]. The objective of this case presentation was to examine the importance of prompt diagnosis, treatment, and counseling to avoid complications. This case emphasizes the value of all-encompassing management techniques in treating DS successfully. It also highlights the significance of patient education, good oral hygiene, and focused therapy in producing favorable results.

## Case presentation

The 72-year-old male patient arrived complaining primarily of burning in his palate and trouble chewing food for one week. For the last week, there has been a burning sensation in the maxillary arch. His history indicated that he was fully edentulous and denture wearer for five years. The patient used dentures 14-18 hours daily for five years. He removed his dentures at night and used them during the day. Based on a history taken, the patient also revealed that the patient wore the denture at night sometimes. An unsightly odor emanated from an ill-fitting maxillary denture that was discolored or stained from the accumulation of food particles, bacteria, and other debris. The patient had no systemic history.

**Figure 1:**
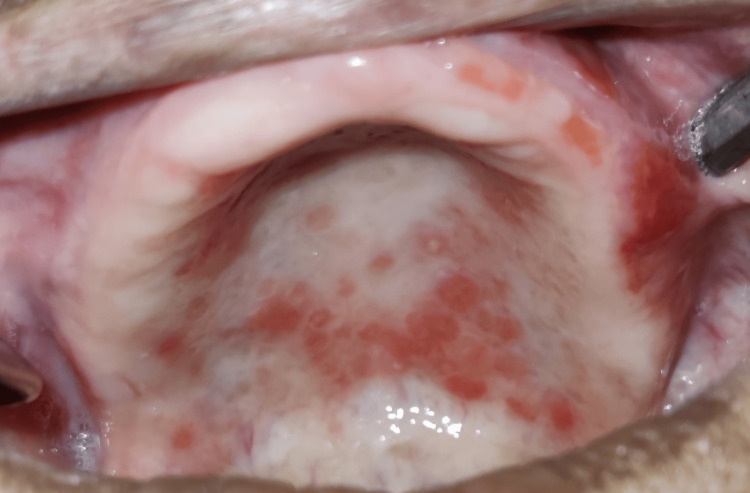
Clinical photograph of the entity showing diffuse erythema over the hard palate Clinical photograph of the entity showing diffuse erythema over the hard palate

Erythematous ulcerations were seen over the hard palate during an intraoral examination, as seen in Figure 1. There was discomfort felt when palpated. Examining the dentures revealed that the teeth's occlusal surfaces had worn away. When the denture's fitting surface was inspected, it was discovered that the patient had fixed it and was using household adhesive to keep the denture in place. Based on clinical presentation, DS was given as a clinical diagnosis. Any allergic reaction and contact dermatitis were considered as differential diagnosis. A smear was not obtained for histology because the patient did not agree to the same.

The patient was told to completely stop using dentures. A diode laser with a wavelength of 980 nm was used on the patient to ablate the palatal lesion for 15 seconds at a low power of 2 watts. Topical local anesthesia was applied to the palatal area. The oral mucosa's inflammatory regions were specifically targeted by the laser. For 15 seconds, each palatal lesion was ablated. There was only one scheduled session, and it lasted a few minutes. It is recommended to patients to practice proper oral hygiene.

Additionally, the patient was instructed to apply ozonated olive oil (ADC Inc. DentozoneIndia) topically three to five times a day until the lesion healed fully. In order to guarantee a clean application area and to get rid of any food particles, the patient was instructed to properly rinse their mouth. He was instructed to apply the ozonated oil directly to the palatal region using a cotton swab or ball after isolating the area as much as possible. If using a dropper, apply a few drops to the palatal region and use a clean finger or cotton swab to disperse them uniformly. In order to give the oil time to absorb into the tissues, the patient was instructed to keep the mouth open. Moreover, wait at least 20 to 30 minutes after application before eating or drinking anything to guarantee the oil stays in contact with the tissue and has time to absorb.

**Figure 2:**
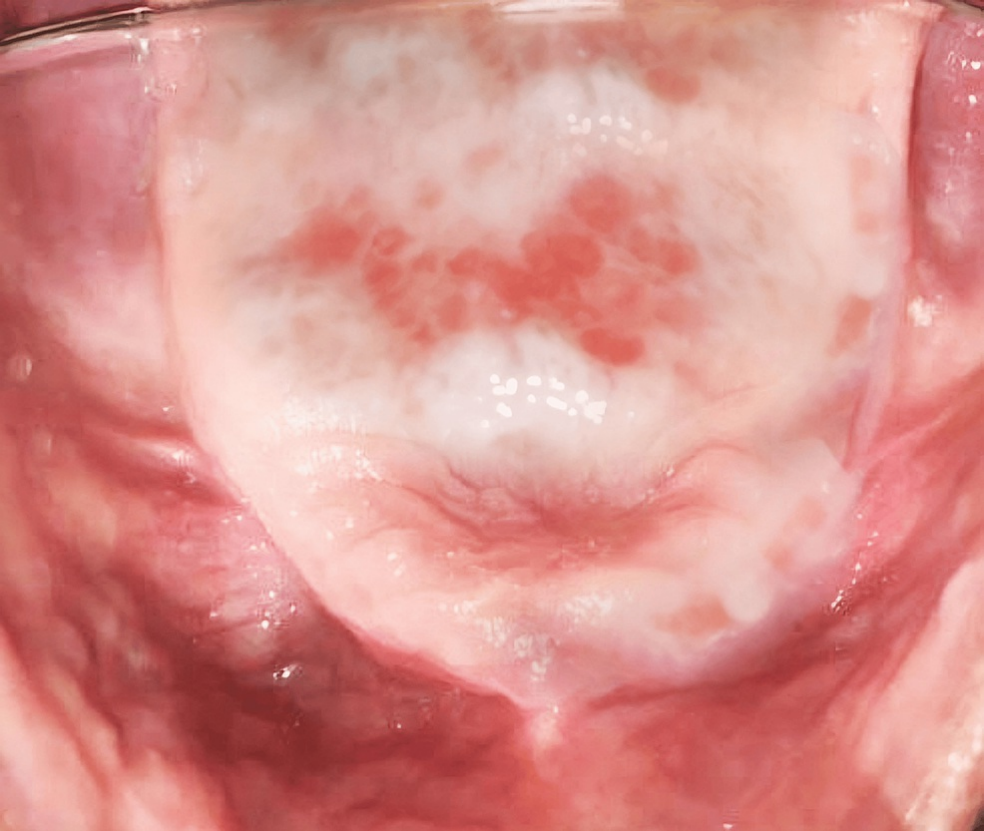
Patient's third-day follow-up photograph of the entity showing reduction in erythema. The photograph is taken with the help of occlusal mirror Patient's third-day follow-up photograph of the entity showing reduction in erythema

The patient reported on the third and fifth day for follow-up. The patient received instructions on how to use dentures and advice on the value of maintaining good oral and denture hygiene. There was a progressive improvement in the lesions' resolution and symptoms. Figure 2 displays the patient's third-day follow-up, which demonstrates a reduction in erythema.

**Figure 3:**
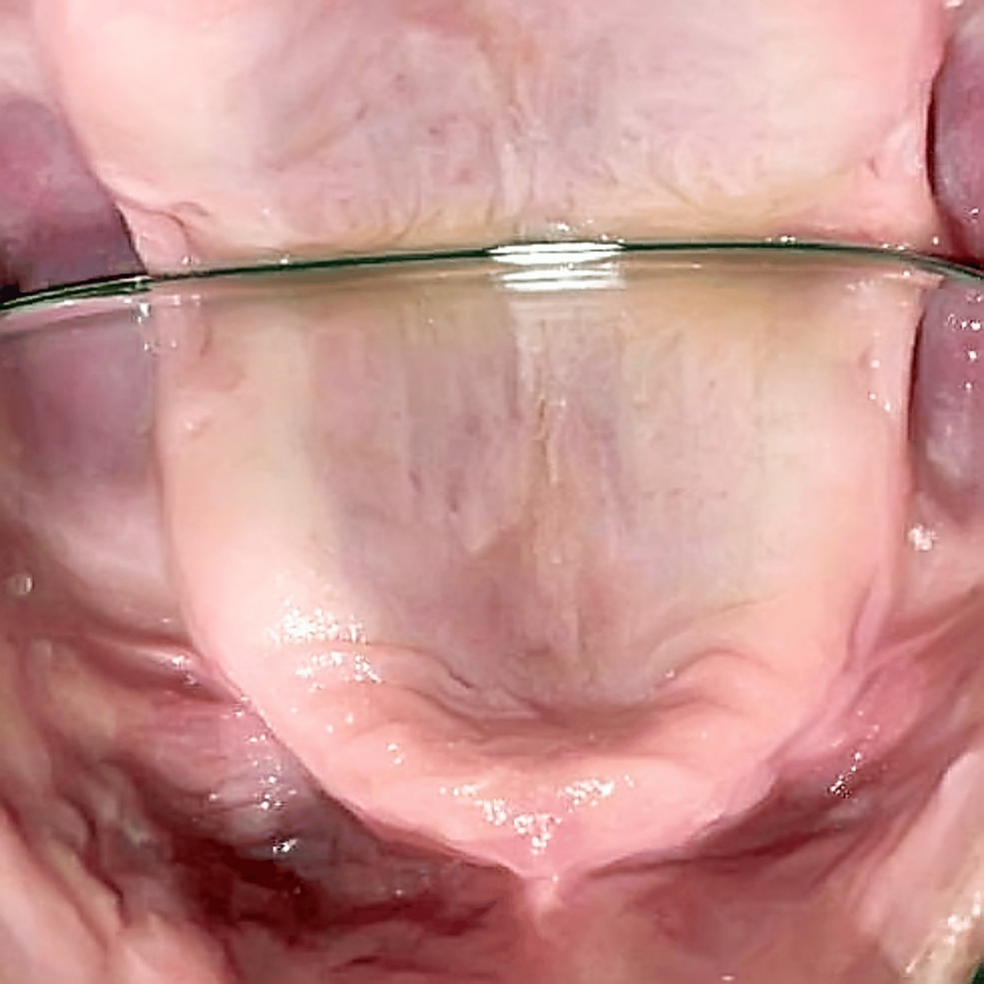
Patient's sixth-day follow-up photograph of the entity showing full resolution of the entity. The photograph is taken with the help of occlusal mirror Patient's sixth-day follow-up photograph of the entity showing full resolution of the entity

Figure 3 illustrates the full resolution of the lesion six days later. The patient was counseled to get a new prosthesis and told to practice good dental hygiene. The patient was told to follow-up regularly.

In order to treat DS effectively, patient education is essential. To avoid the accumulation of plaque, germs, and fungi that might lead to DS, dentures must be cleaned properly. The patient was told to take off their dentures and give them a thorough brushing with a soft-bristled brush and a denture cleanser without any abrasives after meals. Additionally, advised to soak dentures in a denture-cleaning solution for the entire night in order to aid with disinfection. Reminding patients to thoroughly clean their mouths before re-adjusting their dentures is important. This involves using a soft-bristled toothbrush or a moistened gauze pad to clean the palate, gums, and natural teeth, if any are present. Maintaining good dental hygiene might lessen the growth of microbes that worsen stomatitis. Denture-free periods of time may help patients with DS by allowing their oral tissues to breathe and heal. In order to accelerate healing and lessen inflammation, patients should be advised to rinse their mouths with lukewarm seawater or an antiseptic mouthwash during these times. Patients wearing dentures must see their dentist on a regular basis to evaluate their oral health and identify any early warning indications of stomatitis or other issues. It is advisable to counsel patients to make routine appointments with their dentist, who can offer expert cleanings, denture adjustments, and advice on good oral hygiene. Sustaining immune system function and sustaining dental health depend on eating a healthy diet and staying hydrated. Patients ought to get advice on the value of eating a well-balanced diet. Patients should be informed about the things that can make DS more likely, like smoking, improperly managed diabetes, wearing dentures that are too small, and poor oral hygiene. Promoting patients to take care of these risk factors can aid in preventing stomatitis from recurring. Patients should be informed about the things that can make DS more likely, like smoking, improperly managed diabetes, wearing dentures that are too small, and poor oral hygiene. Promoting patients to take care of these risk factors can aid in preventing stomatitis from recurring. Dental practitioners can enable patients to actively participate in controlling their DS and preserving their best oral health by highlighting these hygiene behaviors and offering thorough patient education.

## Discussion

Literature review

Complete and partial removable dentures are linked to the mucosal condition known as DS. A brief overview of the literature on the subject was provided in the 2021 publication by Sartawi et al., with a focus on current systematic reviews' examination of DS treatment procedures. This paper began with a broad overview of the literature, followed by a summary of the most current systematic reviews on DS treatment protocols. Six primary treatment protocols were derived from the collection of 15 systematic reviews. The most recent solutions for treating DS were known to dentists, which aided in thorough treatment planning. Nevertheless, more research is necessary before the more recent denture disinfection techniques can be advised [18].

Da Costa et al. [19] examined five randomized clinical studies that used microwave disinfection to treat DS, seven years after Khiyani et al. [20]. In treating DS, they reported that the 650 W treatment, applied once a week for 14 days, produced higher cost-effect outcomes, was more effective than topical miconazole and as effective as 0.2% chlorhexidine, 0.02% sodium hypochlorite, and topical nystatin. Santos Sousa et al. [21] investigated how antifungal medication and microwave disinfection affected the levels of *Candida *and the outward signs of DS. As a successful antifungal therapy for DS, they suggested microwave disinfection of entire dentures. To validate such data, they did, however, call for more well-planned research. During a 90-day follow-up period, they found that the groups that received topical nystatin medication with microwave disinfection experienced a significant decrease in *Candida* levels and DS frequency. Another technique for sanitizing denture lining materials has been mentioned: microwave irradiation. According to a different systematic evaluation, microwave therapy was noticeably superior to topical miconazole treatment for people who wear dentures [18].

There is also photodynamic therapy for denture decontamination. Using a photosensitizing substance that is activated by light with the right wavelength is the basis of the process. Free radicals and reactive oxygen are created during this interaction in the presence of oxygen, which damages and kills *Candida *cells. Kellesarian et al. [22] evaluated the efficiency of photodynamic treatment in the disinfection of acrylic surfaces of bacteria on acrylic specimens where one randomized control trial indicated that photodynamic therapy was comparable to antifungal medicines for denture decontamination. Nonetheless, the authors suggested that further carefully controlled clinical trials are necessary because the behavior of photodynamic treatment is still uncertain. In a systematic study, Davoudi et al. [23] examined low-level laser therapy (LLLT) in relation to other treatment plans. Four moderately strong scientific papers introduced LLLT as a means of denture disinfection. It has demonstrated a significant role in the clinical treatment of DS and has been effective in reducing the colony-forming unit (CFU/mL) of *Candida albicans* and relieving the inflammation of the soft tissues caused by DS, all without causing any noteworthy side effects [18].

Ozone therapy's effective antibacterial action in both gaseous and aqueous forms made it highly respected in the dentistry industry [24]. DS, primarily caused by *Candida albicans*, was one of the most prevalent problems related to wearing dentures. The study aimed to evaluate and contrast the effects of mouthwash containing chlorhexidine and ozonated water on the healing process of intraoral inflammations linked to complete dentures. Screening was conducted to determine whether any coexisting DS or *Candida albicans* was present in 50 middle-aged male patients who were entirely edentulous and wore complete dentures. Two groups of patients were randomly assigned. Patients in group I received oral hygiene measures (OHM) and mouthwash containing chlorhexidine, whereas patients in group II received OHM and mouthwash containing ozonated water. At every visit, starting on the first day of treatment and continuing every seven, 14, and 30 days later, all patients had their levels of inflammation, pain intensity, and oral candidiasis count evaluated. Using both ozonated water and chlorhexidine mouthwash resulted in a considerable reduction over time in the area and severity of inflammation as well as the pain grade. There were negligible variations between the usage of ozonated water and mouthwash containing chlorhexidine. The number of colonies decreased significantly with time with respect to *Candida-*forming units (CFU), while there was no discernible difference between the two groups. The area and intensity of inflammation as well as the pain threshold were both significantly reduced by ozonated water. Ozonated water could be used as a substitute mouthwash because of its antifungal properties [24].

Discussion

In this case study, one visit laser therapy and topical application of ozonated oil was done. There isn't a general agreement for treating this condition that is widely accepted. On the patient's third-day follow-up, there was reduction in erythematous ulcerations on the palate. On the sixth-day follow-up, there was complete resolution of the condition. Table 1 summarizes the clinical observations at each visit. There is no set course of treatment for patients with DS because the etiology of the condition is so complex. Numerous suggestions have been suggested, including the use of disinfectant substances and antifungal medications, regular cleaning of the denture, and replacement.

**Table 1:** A table summarizing clinical observations at each visit

Clinical observation	Initial stage	Third-day follow-up	Sixth-day follow-up
	Multiple erythmatous ulcerations on the palate region	Reduction in erythema by 40%-50%	Complete resolution of the condition

There are various advantages of using laser therapy in such cases. With laser therapy, damaged tissues can be precisely targeted while sustaining the least amount of harm to nearby healthy tissues. Due to their antibacterial qualities, lasers can successfully lower or eradicate the microbial burden, which includes *Candida albicans*, the main pathogen linked to DS. Because laser therapy has biostimulatory effects that increase blood flow and cellular activity, it can facilitate speedier healing and tissue regeneration. By lowering pain and inflammation brought on by DS, laser therapy's anti-inflammatory qualities might help patients feel more comfortable. Compared to surgical procedures, laser treatment is less invasive, which means that there are fewer risks and faster recovery periods. Due to the high cost of laser equipment and the requirement for specialized training, one of laser therapy's main drawbacks is its potential expense. Additional constraints encompass availability, technical proficiency, and variable efficiency. Possible adverse effects arise if laser settings are not properly adjusted, oral tissues may sustain heat damage that results in burns or necrosis. Following laser treatment, some patients may have brief periods of discomfort or sensitivity. Patients who are photosensitive due to medical disorders or drugs should not receive laser therapy since this may intensify their light sensitivity. To avoid possibly stimulating malignant cells, laser therapy should be avoided in locations where known or suspected malignancies exist. Due to insufficient information on laser therapy's safety in expectant mothers, it is usually not recommended to be used during pregnancy. Before beginning laser therapy, patients with pacemakers should have a thorough evaluation because some lasers can interact with the device [25-29].

Strong antibacterial qualities of ozone enable it to efficiently eradicate bacteria, fungi, and viruses, including the main pathogen linked to DS, *Candida albicans, f*urthermore having anti-inflammatory qualities that lessen pain. Ozone therapy helps hasten the healing process by increasing blood flow and oxygenation to the injured tissues. Since topical ozone administration is a noninvasive therapy, it is a good choice for patients who would rather forego more intrusive procedures. Some patients may not have access to ozone therapy in all dental practices due to its limited availability. Ozone has a brief half-life, which means that in order to reap long-term advantages, its antibacterial effects may be momentary and require frequent administrations. Although short-term studies have demonstrated the potential of ozone therapy, comprehensive long-term study is needed to properly understand its safety and efficacy over extended periods of time. Allergy responses and tissue inflammation are possible adverse effects. Ozone therapy should be avoided by patients with severe respiratory disorders, such as asthma or chronic obstructive pulmonary disease, as ozone can irritate the respiratory tract. Pregnant women should generally avoid ozone therapy as its safety has not been fully proved during pregnancy. To avoid negative responses, ozone therapy should not be administered to patients who have a documented allergy to ozone. Ozone therapy may need to be avoided by those with specific autoimmune conditions since it may make their condition worse [30-34].

The reported case falls within Newton classification type 2 DS. It was a persistent illness brought on by microbiological agents linked to wear-related mechanical damage from wearing dentures all day and night as well as inadequate oral hygiene. The regular maintenance of proper dental hygiene is essential to preventing this illness. Oral health providers must provide accurate patient counseling and guidance. The following skills must be developed to treat DS: cleaning and sanitizing dentures, wearing them as prescribed by a dentist, mechanical plaque control, and using mouthwashes such as chlorhexidine. Adjust or replace the faulty dentures [35,36].

The majority of patients are asymptomatic and are diagnosed based on a routine clinical examination; clinical features of DS include burning sensation, diffuse erythema of the involved mucosa with hyperemic points, and halitosis [2,7,10,14]. Treatment for DS varies depending on the type of predisposing factors involved and the clinical types [16]. An extensive examination of the oral cavity, including looking at the soft and hard palate and examining the buccal mucosa in those who have had their dentures removed, is usually a good place to start. Predisposing variables are determined and addressed. The kind, intensity, and chronicity of the infection are also addressed. It seems that maintaining proper dentures and dental hygiene should be the most crucial rule to be followed. Antifungal medications are a successful treatment for DS, and full lesion resolution happens in 12 to 14 days [34-38]. Antifungal agent use has certain disadvantages as well, such as the need for continuous use for at least two to four weeks, irregular dosing that might lead to *Candida* species resistance, and the possibility of lesion recurrence. The most widely used disinfectant, chlorhexidine, shouldn't be used with nystatin since it may reduce the latter's ability to fight fungal infections [25]. Antifungal medications should thus be saved for more severe lesions affecting other oral mucosal surfaces and in individuals who are severely disabled [38].

Topical treatments can be used to specifically manage candidiasis; if the condition is not under control, systemic medication should be used as a form of treatment. Similarly, combining topical medicines with systemic treatments can be beneficial for patients with persistent infections. This might permit using systemic drugs for a shorter period or at a lower dose [39]. Care decisions are entirely based on the kind, severity, and duration of the condition. It is advised that the course of antifungal therapy be continued for at least twice as long as the cessation of clinical signs and symptoms. Taste, ease of administration, texture (which may contain alcohol or sucrose), possible drug sensitivity or resistance, and cost should all be taken into account when choosing an antifungal prescription. Tablets intended for oral dissolution may not dissolve as well in patients with dry mouths. For patients who have a dry mouth in particular, oral liquid suspensions might be a good option [36,37]. In both immunocompetent and immunocompromised hosts, fluconazole works against a wide range of fungal infections. Tacrolimus and cyclosporine A have synergistic efficacy in vitro against *Aspergillus*, *Candida*, and* Cryptococcus* neoformans when combined with azoles. The synergistic impact of tacrolimus and fluconazole against *Candida albicans* biofilms is credited to calcineurin inhibition [39,40].

As a first line of treatment, topical therapy with antifungals should be administered by soaking dentures in any antiseptic solution. Additionally, as a result, the number of microbial colonies on the denture's surface decreases [11]. Treatment should also be given for underlying systemic diseases like diabetes mellitus. Advise patients on corticosteroids about the safety measures to be followed when using the drug, such as washing their mouths after breathing the drug, among other things [5,41]. Although it can be connected to any intraoral candidiasis, angular cheilitis is one of the most commonly observed conditions linked to DS. Tissue conditioners are also used to enhance denture adaption, permit denture-bearing tissues to heal, and lower the number of *Candida* colonies, according to a study by Iqbal et al. [36]. In the present case, the patient was instructed to remove the denture and was given information about using topical ozonated oil to gently apply to the tissue before the fabrication of a new denture, as the denture was in poor condition and tissue conditioner was not implemented.

There are other studies available on how well ozone works to treat cutaneous infections. It has been proven that ozone therapy affects the growth of bacteria and their structural modifications, including *Salmonella*, *Escherichia coli*, *Staphylococcus aureus*, and *Bacillus subtilis* [12-14]. Through the disk diffusion method, the antifungal activity of ozonized oil (OZ) against yeasts associated with onychomycosis was investigated [12]. OZ may be a novel treatment option for the management of biofilm in DS cases, according to a study that assessed the impact of OZ on the oral levels of *Candida *species in these individuals [12]. Because topical ozonated oil therapy has antibacterial qualities, anti-inflammatory effects, healing properties, relieves oxidative stress, and improves tissue circulation, we used it in the current case [13]. Laser therapy seems to be more obliging than antifungal medication. In addition to aiding in the ablation of the epithelial surface contaminated with *Candida*, a laser beam also helps to avoid inflaming the nearby normal mucosa [30]. Since the provided laser energy looks to be bactericidal and viricidal, there will be no need for postoperative prescriptions of antibiotics or nonsteroidal anti-inflammatory medicines. This could stop germs from reinfecting or spreading [41-43]. Adequate pain management is aided by lasers' neuron sealing effect [43]. Clinically, diode laser treatment was administered for type 2 DS in our instance. Within a week, the patient's condition had fully resolved. Up to six months of follow-up, exams revealed no infection recurrence in the patient. Treatment for DS and papillary hyperplasia is identical. However, more active care will be necessary if there is tissue excess that may not mend with conservative measures. Depending on the severity of the ailment and the clinical presentation, different practitioners will prescribe different courses of treatment [19]. Electrosurgery or cryotherapy is advised when intense laser excision is the clinical manifestation [44]. Chlorhexidine mouthwash has been used topically for small, localized lesions [45].

If adequate denture care and oral hygiene habits are not followed, DS may return even after a successful initial course of treatment. Within a year of treatment, recurrence rates have been shown in studies to range from 15% to 50% [46]. DS can result in problems such oral candidiasis, angular cheilitis, and lesions on the oral mucosa if it is not treated or handled in an ineffective manner. The patient's quality of life may be impacted by these issues, which may call for further treatments [47]. DS over time can worsen masticatory function, cause discomfort in the mouth, and lower quality of life when it comes to dental health. To monitor the condition and prevent problems, it is imperative to schedule regular follow-up sessions with a dentist [1]. Studies have indicated an association between systemic illnesses like respiratory infections and cardiovascular disease and oral candidiasis, which includes DS. To prove a clear-cut causal link, additional research is necessary [48].

In order to treat DS effectively, patient education is essential. To avoid the accumulation of plaque, germs, and fungi that might lead to DS, dentures must be cleaned properly. The patient was told to take off their dentures and give them a thorough brushing with a soft-bristled brush and a denture cleanser without any abrasives after meals and was advised to soak dentures in a denture-cleaning solution for the entire night in order to aid with disinfection. Reminding patients to thoroughly clean their mouths before readjusting their dentures is important. This involves using a soft-bristled toothbrush or a moistened gauze pad to clean the palate, gums, and natural teeth, if any are present. Maintaining good dental hygiene might lessen the growth of microbes that worsen stomatitis. Denture-free periods of time may help patients with DS by allowing their oral tissues to breathe and heal. In order to accelerate healing and lessen inflammation, patients should be advised to rinse their mouths with lukewarm seawater or an antiseptic mouthwash during these times. Patients wearing dentures must see their dentist on a regular basis to evaluate their oral health and identify any early warning indications of stomatitis or other issues. It is advisable to counsel patients to make routine appointments with their dentist, who can offer expert cleanings, denture adjustments, and advice on good oral hygiene. Sustaining immune system function and sustaining dental health depend on eating a healthy diet and staying hydrated. Patients ought to get advice on the value of eating a well-balanced diet. Patients should be informed about the things that can make DS more likely, like smoking, improperly managed diabetes, wearing dentures that are too small, and poor oral hygiene. Promoting patients to take care of these risk factors can aid in preventing stomatitis from recurring. Patients should be informed about the things that can make DS more likely, like smoking, improperly managed diabetes, wearing dentures that are too small, and poor oral hygiene. Promoting patients to take care of these risk factors can aid in preventing stomatitis from recurring. Dental practitioners can enable patients to actively participate in controlling their DS and preserving their best oral health by highlighting these hygiene behaviors and offering thorough patient education.

## Conclusions

 A 72-year-old male patient complaining about burning in his palate was diagnosed with DS. Management was done by topical laser therapy and topical ozonated olive oil. The third day's follow-up visit revealed a progressive healing of the lesions and symptom relief. The lesion fully resolved on the sixth day. This case study highlights the effectiveness of comprehensive management techniques, including patient education, good oral hygiene practices, topical ozone therapy, and diode laser therapy in successfully treating DS. To properly identify and treat the illness, a thorough history and examination are essential. The patient's symptoms significantly improved and the inflammatory lesions resolved as a result of receiving education on denture use, laser application, and ozonated oil application. To effectively treat DS, a comprehensive management plan is crucial, and the case highlights the necessity of both compliance from patients and clinician intervention. To optimize the therapy of this prevalent oral illness, more investigation into modalities of treatment and preventive strategies is necessary, including clinical investigations. All things considered; this instance shows how customized treatment plans that target the underlying causes of DS can lead to positive results.
